# Acute Pancreatitis Associated With Anaphylactic Shock: A Case Report and Literature Review

**DOI:** 10.7759/cureus.107221

**Published:** 2026-04-17

**Authors:** Gauthier Leclercq, Fabien Guerisse

**Affiliations:** 1 Emergency Department, Centre Hospitalier Universitaire (CHU) Charleroi-Chimay, Charleroi, BEL

**Keywords:** acute pancreatitis, anaphylactic shock, anaphylaxis, case report, hypotension, lipase elevation, literature review, pancreatic involvement

## Abstract

Acute pancreatitis is most commonly caused by gallstones or alcohol abuse, although rare etiologies such as drug-induced anaphylaxis have been described. Pancreatic involvement during anaphylactic shock remains exceptional and is likely underdiagnosed. We report the case of a 58-year-old woman who developed acute circulatory shock shortly after the topical application of a hemorrhoidal ointment (TriAnal). She presented with hypotension, tachycardia, pruritus, and erythematous urticarial lesions. Laboratory investigations revealed elevated serum lipase and tryptase. Abdominal computed tomography demonstrated peripancreatic fat stranding and focal pancreatic enlargement, consistent with mild edematous pancreatitis (Balthazar grade C). The patient was treated promptly with epinephrine, corticosteroids, fluid resuscitation, and vasopressor support, leading to rapid clinical and biochemical improvement. Pancreatic injury in the context of anaphylactic shock may result from a dual mechanism involving ischemia due to hypoperfusion and immune-mediated inflammation triggered by the release of vasoactive mediators. This form of pancreatitis is often clinically silent, with abdominal pain that may be absent or transient, making biological and radiological findings essential for diagnosis. Early hemodynamic stabilization remains the cornerstone of management, and pancreatic abnormalities generally resolve quickly. Although rare, acute pancreatitis associated with anaphylactic shock is probably underrecognized, and clinicians should consider this diagnosis in cases of severe anaphylaxis, particularly in the presence of digestive symptoms or unexplained elevations of pancreatic enzymes, as prompt recognition and appropriate management are key to ensuring favorable outcomes.

## Introduction

Acute pancreatitis is a common inflammatory disease of the pancreas, most frequently caused by gallstones and excessive alcohol consumption. Nevertheless, up to 20% of cases are classified as idiopathic. Among rare etiologies, the association between severe allergic reactions, anaphylactic shock, and acute pancreatitis is seldom described and likely underdiagnosed [1,2].

Anaphylactic shock is characterized by a massive release of inflammatory mediators leading to systemic vasodilation, increased vascular permeability, tissue hypoperfusion, and potentially multiorgan failure. In this context, pancreatic involvement remains exceptional in the literature and is mainly reported through isolated case reports [3,4].

We report a case of acute pancreatitis that presented to the emergency room and review the literature to discuss the pathophysiological mechanisms involved, the histopathological characteristics, and the clinical implications.

## Case presentation

A 58-year-old woman was admitted to the emergency room for acute circulatory shock occurring shortly after the topical application of a hemorrhoidal ointment, TriAnal (triamcinolone acetonide and lidocaine hydrochloride).

Her medical history included treated breast cancer, arterial hypertension, impaired glucose tolerance, hypercholesterolemia, and regular alcohol consumption. No previous allergic history was reported.

Approximately 30 minutes after the application of the ointment, the patient developed acute epigastric pain associated with diarrhea. Upon arrival of the emergency response team, she was hypotensive, she had tachycardia at 111 beats per minute, dyspneic but had a good oxygen saturation, and presented diffuse pruritic skin eruptions. The epigastric pain had receded at that time. Immediate emergency response included intramuscular injection of epinephrine (0.5 mg) and intravenous injection of methylprednisolone (125 mg).

Upon admission to the emergency room, hypotension persisted at 71/48 mmHg, along with intense pruritus. Physical examination revealed generalized erythema with widespread urticarial plaques. Fluid resuscitation was initiated (total of 3 liters of crystalloids), and the patient was subsequently admitted to the intensive care unit with norepinephrine infusion to mitigate vasoplegic shock.

Laboratory findings at admission are summarized in Table 1. Blood tests showed elevated lipase levels with normal liver function, as well as an increase in tryptase levels, confirming an anaphylactic mechanism. All other laboratory tests were within normal limits.

**Table 1:** Laboratory findings at admission CRP: C-reactive protein; eGFR: estimated Glomerular Filtration rate; CKD-EPI: Chronic Kidney Disease – Epidemiology Collaboration Equation

Parameter	Patient Value	Unit	Reference Range
White Blood Cells	7.34	×10³/µL	4.00–10.00
CRP	7	mg/L	<5
Lipase	420	U/L	8–78
Creatinine	0.96	mg/dl	0.54-1.12
eGFR (CKD-EPI)	65.0	mL/min/1.73m²	> 60.0
Tryptase	25.7	µg/L	<8.40

Arterial blood gas analysis is shown in Table 2, revealing a mild metabolic acidosis and elevated lactate.

**Table 2:** Arterial blood gas analysis PaCO₂: partial pressure of carbon dioxide; PaO₂: partial pressure of oxygen.

Parameter	Patient Value	Unit	Reference Range
pH	7.33	—	7.35–7.45
PaCO₂	37	mmHg	32–45
PaO₂	72	mmHg	83–108
Bicarbonate	19.5	mEq/L	21.0–28.0
Lactate	3.2	mmol/L	0.4–0.8

**Figure 1:**
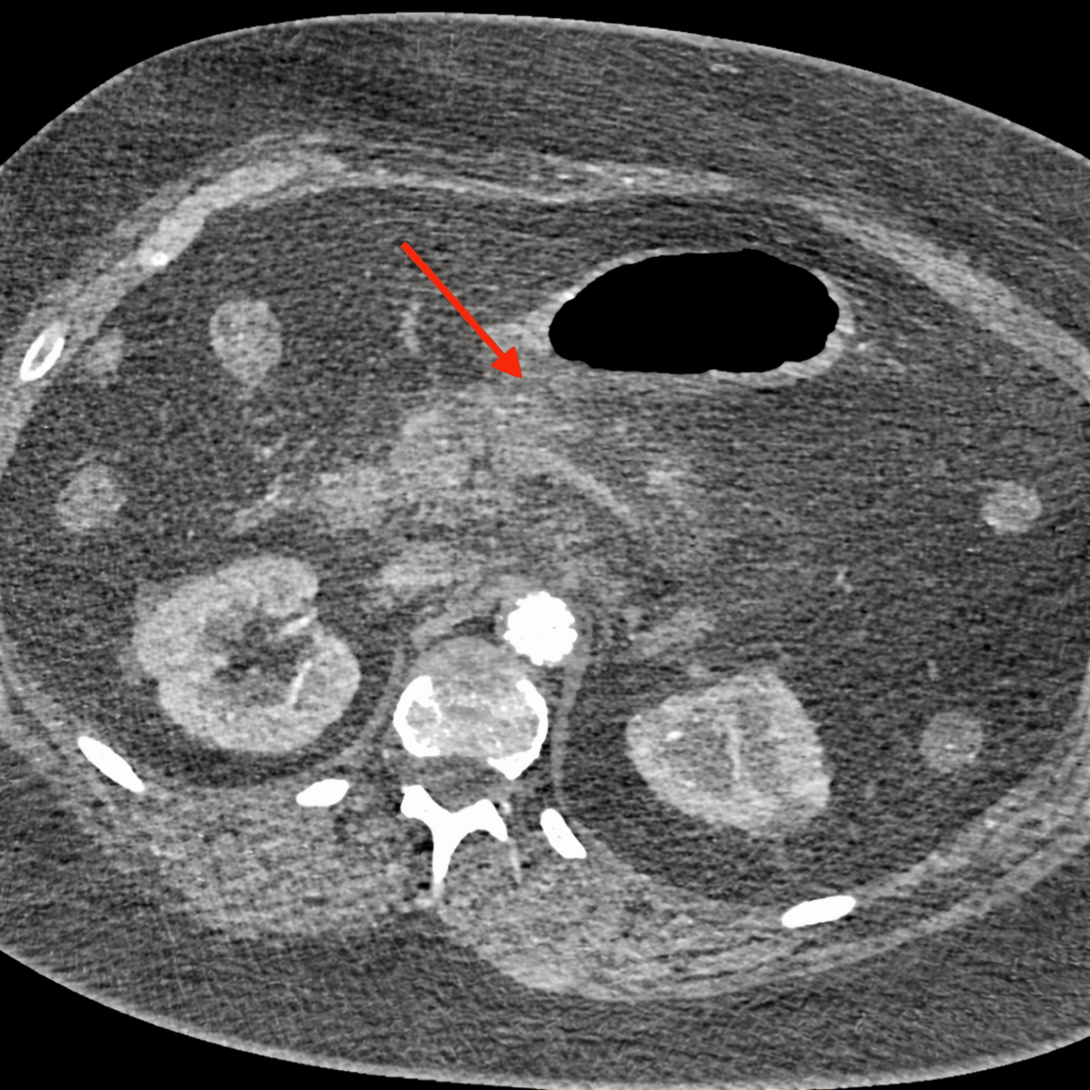
Aortic CT angiography The red arrow indicates peripancreatic fat stranding and focal enlargement of the pancreatic body, consistent with edematous acute pancreatitis, Balthazar grade C.

Abdominal CT demonstrated mild peripancreatic fat stranding and focal enlargement of the pancreatic body, consistent with edematous acute pancreatitis (Balthazar grade C), as illustrated in Figure 1.

The final diagnosis was vasoplegic shock secondary to drug-induced anaphylaxis, associated with mild acute pancreatitis. Clinical and biological evolution was rapidly favorable, with normalization of serum lipase within 24 hours. The patient was discharged from the intensive care unit the following day.

## Discussion

Acute pancreatitis associated with anaphylaxis

Several cases of acute pancreatitis following anaphylactic reactions, particularly food-induced, have been reported. Wiese et al. described a case of acute pancreatitis occurring approximately 12 hours after food-induced anaphylaxis, without an identifiable classical etiology [3]. Similarly, Toki et al. reported a pediatric case of biologically confirmed acute pancreatitis associated with cashew nut-induced anaphylaxis, notably without typical abdominal pain [4]. These observations are in line with our clinical case study, where initial digestive symptomatology was brief and quickly hidden by the hemodynamic and cutaneous profile, implying that the diagnosis of pancreatitis relies on the laboratory and radiology tests. Additional reports support the association between anaphylaxis and pancreatic injury, suggesting that digestive symptoms may be transient or masked by the severity of the systemic reaction [3,4].

Drug-induced hypersensitivity and pancreatitis

Severe drug hypersensitivity reactions have also been implicated in acute pancreatitis. An anaphylactic reaction associated with ranitidine administration was reported in a patient hospitalized for acute pancreatitis [5]. Pharmacovigilance alerts have described cases of acute pancreatitis associated with severe hypersensitivity reactions to antidiabetic agents such as saxagliptin [6]. In our case, the close temporal relationship between topical drug exposure and the onset of symptoms, coupled with elevated serum tryptase, strongly supports a drug-induced immuno-allergic mechanism.

Role of shock and hypoperfusion

Histopathological studies have demonstrated that severe shock states may induce pancreatic ischemic injury [7]. Cases of acute pancreatitis have also been reported following anaphylactic shock due to toxic exposures, including multiple hymenoptera stings [8]. These findings suggest that pancreatic hypoperfusion, combined with inflammatory mediator release, plays a critical role in pancreatic injury during shock. Our case illustrates an edematous acute pancreatitis occurring in the setting of severe anaphylactic shock, a rare but physiopathologically coherent association.

Pancreatic ischemia and histopathological features

The pancreas is particularly susceptible to ischemic injury. Experimental and clinical studies have shown that even transient hypoperfusion may result in pancreatic damage [9]. Kovacs et al. provided a detailed histopathological description of pancreatitis associated with shock states, reporting acinar cell necrosis, interstitial edema, vascular congestion, and microthrombosis, with minimal inflammatory infiltrate compared to classical acute pancreatitis [7]. These findings suggest that ischemia rather than primary enzymatic activation is the dominant mechanism in shock-related pancreatitis. Ischemia-reperfusion injury may further exacerbate pancreatic damage through oxidative stress and endothelial dysfunction [10].

Immuno-allergic contribution

Massive mediator release (histamine, cytokines, leukotrienes) that occurs simultaneously with the anaphylaxis shock leads to increased capillary permeability and tissue edema. This inflammatory cascade may directly affect pancreatic microcirculation, amplifying ischemic injury and triggering pancreatic inflammation [3,4]. The elevated serum tryptase in our patient supports this combined ischemic and immunological mechanism. These observations suggest that pancreatic involvement in the context of anaphylactic shock results from a dual mechanism combining ischemia and immune-mediated inflammation, and that such involvement may be transient and reversible if the triggering cause is promptly identified and treated. The rapid normalization of lipase and the favorable clinical course observed in our case are consistent with the literature, confirming that pancreatic involvement may serve as a marker of systemic severity and that recovery is possible with appropriate management.

Diagnostic challenges

Shock-related pancreatitis is frequently clinically silent. Abdominal pain may be absent, transient, or masked by shock-related symptoms, sedation, or altered mental status [7]. Consequently, diagnosis often relies on biological abnormalities and imaging rather than clinical presentation alone, as observed in our patient.

Implications for clinical management

Management of the patient primarily relies on rapid correction of the shock state, including appropriate fluid resuscitation and vasopressor support to restore tissue perfusion [11]. Current international guidelines emphasize early hemodynamic optimization, close monitoring in intensive care, and early enteral nutrition when feasible [11-13]. In shock-related pancreatitis, pancreatic involvement often reflects systemic severity rather than primary pancreatic disease, explaining the generally favorable evolution when hemodynamic instability is promptly corrected [7,14].

## Conclusions

Acute pancreatitis associated with anaphylactic shock is a rare but likely underdiagnosed entity. This case highlights the importance of considering pancreatic involvement in patients with severe anaphylaxis, especially when early digestive symptoms or unexplained enzyme elevations are present. Early recognition and prompt hemodynamic management are essential to ensure favorable outcomes.
